# Associations of *HMGA2* single-nucleotide polymorphisms with clinicopathological features of tongue squamous cell carcinoma in Taiwanese men with distinct lifestyle-related risk factors

**DOI:** 10.7150/jca.139096

**Published:** 2026-09-11

**Authors:** Yi-Chieh Yang, Chia-Hwa Lee, Yi-Fang Ding, Chiao-Wen Lin, Lun-Ching Chang, Yu-Ching Wen, Shun-Fa Yang, Ming-Hsien Chien

**Affiliations:** 1 School of Oral Hygiene, College of Oral Medicine, Taipei Medical University, Taipei, Taiwan.; 2 School of Medical Laboratory Science and Biotechnology, College of Medical Science and Technology, Taipei Medical University, Taipei, Taiwan.; 3 Department of Otolaryngology, Wan Fang Hospital, Taipei Medical University, Taipei, Taiwan.; 4 Department of Otolaryngology, Chung Shan Medical University Hospital, Taichung, Taiwan.; 5 Institute of Oral Sciences, Chung Shan Medical University, Taichung, Taiwan.; 6 Department of Mathematics and Statistics, Florida Atlantic University, Boca Raton, FL, USA.; 7 Department of Urology, Wan Fang Hospital, Taipei Medical University, Taipei, Taiwan.; 8 Institute of Medicine, Chung Shan Medical University, Taichung, Taiwan.; 9 Department of Medical Research, Chung Shan Medical University Hospital, Taichung, Taiwan.; 10 Graduate Institute of Clinical Medicine, College of Medicine, Taipei Medical University, Taipei, Taiwan.; 11 Pulmonary Research Center, Wan Fang Hospital, Taipei Medical University, Taipei, Taiwan.; 12 Traditional Herbal Medicine Research Center, Taipei Medical University Hospital, Taipei, Taiwan.; 13 TMU Research Center of Cancer Translational Medicine, Taipei Medical University, Taipei, Taiwan.

**Keywords:** oral squamous cell carcinoma, high-mobility group A protein 2, single-nucleotide polymorphism, lifestyle-related risk, aggressive tumor characteristics, male population

## Abstract

**Background:**

Oral squamous cell carcinoma (OSCC) is a predominant malignancy of the oral cavity, with tongue squamous cell carcinoma (TSCC) accounting for a substantial proportion of cases worldwide. High-mobility group A protein 2 (HMGA2) is a nonhistone chromatin-binding factor implicated in aggressive malignant phenotypes of various human cancers. However, the effects of *HMGA2* variants on OSCC susceptibility and tumor clinicopathological aggressiveness remain unclear.

**Methods:**

We examined the associations of functional *HMGA2* single-nucleotide polymorphisms (SNPs) with TSCC susceptibility and aggressive tumor characteristics in Taiwanese men. Four tagging SNPs (rs6581658 A>G, rs10573247 T>del, rs968697 T>C, and rs8756 A>C) were genotyped using a TaqMan allelic discrimination assay. In addition, HMGA2 expression patterns, as well as their associations with disease aggressiveness and patient survival outcomes, were analyzed using multiple independent datasets, including TCGA, CPTAC, and GEO.

**Results:**

Our results indicated no significant association between the four SNPs and TSCC susceptibility. However, carriers of the rs6581658 G allele had significantly increased risks of large tumors (>T2) and advanced clinical stage (stage III or IV) in the dominant model. Moreover, the rs10573247 variant was significantly associated with an increased risk of lymph node metastasis in patients exposed to environmental carcinogens, including betel quid and cigarette smoke. By contrast, the rs968697 variant exerted a protective effect against advanced disease and lymph node metastasis in patients without such environmental exposure. Analyses of clinical datasets pertaining to an American cohort with head and neck squamous cell carcinoma and a Taiwanese cohort with OSCC revealed that HMGA2 expression was upregulated in tumor tissues and was associated with poor prognosis.

**Conclusion:**

These findings suggest potential exposure-stratified association in TSCC and highlight *HMGA2* polymorphisms as promising biomarkers for risk assessment and disease monitoring in male patients with TSCC.

## Introduction

Oral squamous cell carcinoma (OSCC) is the most common malignancy of the oral cavity, accounting for approximately 90% of all types of oral cancer. OSCC predominantly originates in the tongue, where it is referred to as tongue squamous cell carcinoma (TSCC). Poor prognosis in OSCC is largely attributable to its aggressive invasion and metastasis patterns, adverse clinicopathological features, and limited treatment response. OSCC is a complex and heterogeneous disease whose development is influenced by genetic, epigenetic, environmental, and lifestyle factors 1-3. Therefore, identifying reliable genetic biomarkers for TSCC is crucial, and further research is required to determine the roles of candidate genes in TSCC progression.

High-mobility group A protein 2 (HMGA2) is a 160-kb gene located on chromosome 12. As an architectural transcription factor, *HMGA2* contains three AT-hook domains that bind to AT-rich sequences in the minor groove of DNA, which alters the chromatin architecture and modulates the assembly and maintenance of enhancer complexes, ultimately regulating the transcription of numerous genes 4, 5. In addition, HMGA2 functions as a transcriptional coregulator by recruiting other transcription-associated proteins 6. Overexpression of *HMGA2* variants promotes the growth of benign tumors, indicating that HMGA2 promotes cell proliferation and contributes to tumorigenesis 7. Indeed, HMGA2 overexpression has been implicated in tumor aggressiveness, and chemotherapy resistance across human malignancies, including OSCC 8-11. The molecular mechanisms underlying HMGA2-mediated carcinogenesis have been extensively studied 8. For example, HMGA2 interacts with protein phosphatase 4 regulatory subunit 1 to facilitate cell migration and metastasis by activating epithelial-mesenchymal transition (EMT) in non-small cell lung cancer 12. In OSCC, tumorigenesis is further promoted by the histone lysine methyltransferase SMYD3, which enhances *HMGA2* transcription through H3K4me3-mediated epigenetic regulation 13.

Recently, researchers have increasingly investigated the role of genetic risk factors, particularly single-nucleotide polymorphisms (SNPs), in the early prediction and prognosis of OSCC 4. SNPs refer to single-base substitutions, insertions, or deletions that occur at specific genomic loci, with an average frequency of approximately 1 in every 800 base pairs 5. By definition, these variants must be present in at least 1% of the general population. Genome-wide association studies have identified significant associations between height and *HMGA2* SNPs 14. However, molecular genetic evidence regarding the role of *HMGA2*-related SNPs in cancer remains limited. Very few studies have examined the association between *HMGA2* polymorphisms and cancer risk 15-17, and data specific to OSCC remain lacking. In the present case-control study, we investigated the associations of *HMGA2* polymorphisms with TSCC susceptibility and clinicopathological characteristics in Taiwanese men.

## Materials and Methods

### Study cohort

This study included 383 men with TSCC who received treatment at Chung Shan Medical University Hospital, Taichung, Taiwan. The study protocol was approved by the Ethics Committee of Chung Shan Medical University Hospital (approval number: CS1-21151). All participants provided written informed consent before enrollment. They also provided detailed information on lifestyle-related risk factors for cancer—for example, alcohol consumption, cigarette smoking, and betel quid chewing. This study also included 911 anonymized healthy controls randomly selected from the Taiwan Biobank Project. None of these controls had a history of cancer at any site. Patients with oral precancerous conditions were excluded from the control group. Clinical data pertaining to patients with TSCC were retrieved from medical records and included tumor-node-metastasis clinical stage, primary tumor size, lymph node (LN) involvement, distant metastasis status, and tumor histologic grade. All participants included in the control group were male, and the case and control groups were comparable in terms of ethnicity and geographic background, as all participants were Taiwanese individuals recruited from the same population. Therefore, the potential influence of population stratification is expected to be limited.

### Genomic DNA extraction and *HMGA2* SNP selection

Whole blood samples were collected in EDTA-coated tubes and centrifuged at 3,000 rpm to isolate the buffy coat. Genomic DNA was extracted from peripheral blood leukocytes by using a QIAamp DNA Mini Kit (Qiagen, Valencia, CA, USA), following the manufacturer's instructions. DNA purity and concentration were measured using a NanoDrop 2000 spectrophotometer (Thermo Fisher Scientific, Waltham, MA, USA), and the samples were stored at -20°C until genotyping. On the basis of data obtained from the SNPinfo (https://snpinfo.niehs.nih.gov/snpinfo/snpfunc.html) and dbSNP (https://www.ncbi.nlm.nih.gov/snp/) databases, we selected four potentially functional polymorphisms of *HMGA2*: rs6581658 A>G, rs10573247 T>del, rs968697 T>C, and rs8756 A>C. These SNPs were also selected based on functional predictions, minor allele frequency (MAF), linkage disequilibrium (LD) criteria, and prior cancer association studies, as prior studies have demonstrated their association with cancer susceptibility or aggressive tumor characteristics across malignancies 15-20. Based on the dbSNP database, a MAF threshold of > 0.10 was set for the Asian reference population to ensure sufficient statistical power. The MAFs of rs6581658 (A>G), rs10573247 (T>del), rs968697 (T>C), and rs8756 (A>C) in the Asian population were 0.219 (G), 0.391 (T), 0.138 (C), and 0.135 (C), respectively. In cancer cohorts, the rs8756 A>C and rs6581658 A>G polymorphisms have been reported to decrease and increase the risk of brain tumors in Chinese children, respectively 15, 18. The rs968697 T>C polymorphism has been linked to Wilms tumor susceptibility 16, and its CC genotype is associated with a lower frequency of advanced-stage colorectal cancer^19^. Likewise, individuals carrying the TC/CC genotypes of rs968697 exhibit a significantly reduced risk of hepatoblastoma 20. Additionally, the *HMGA2* rs10573247 polymorphism has been implicated in breast cancer susceptibility 17. To ensure independent genetic representation across the *HMGA2* locus and eliminate redundant tagSNPs, pairwise LD analysis was performed. All pairwise LD values among these four variants were below 0.30 (the highest LD was observed between rs10573247 and rs8756, with an LD value of 0.288), indicating that these selected variants represent distinct, non-redundant genetic variations in *HMGA2*. Two of these SNPs—rs8756 A>C and rs10573247 T>del—are located in the 3′ untranslated region (3′ UTR) of *HMGA2*, where they affect microRNA (miRNA) binding and thus modulate gene expression and mRNA stability. By contrast, the other two SNPs—rs6581658 A>G and rs968697 T>C—are located in the 5′ near-gene region, where they modify transcription factor binding and thus regulate *HMGA2* transcription. In the present study, no significant linkage disequilibrium was observed among the selected SNPs (*R*^2^ < 0.8).

### Genotyping of *HMGA2* SNPs

***HMGA2*** SNPs were genotyped using an ABI StepOnePlus RT-PCR system (Applied Biosystems, Foster City, CA, USA) with the following individual TaqMan SNP probes: rs6581658 (assay ID: C__44861231_10), rs10573247 (assay ID: C__63497439_20), rs968697 (assay ID: C___8702860_20), and rs8756 (assay ID: C___1155510_20). The resulting genotypes were analyzed using SDS version 3.0 (Applied Biosystems). Detailed protocols for these procedures have been described elsewhere 21.

### Bioinformatic analyses

Data for this study were obtained from the UALCAN online platform (http://ualcan.path.uab.edu; accessed February 23, 2026) 22, developed by the University of Alabama at Birmingham. This platform was used both to analyze HMGA2 mRNA and protein expression levels in normal and primary tumor tissues and to identify their associations with clinicopathological parameters, including tumor grade, in patients with head and neck squamous cell carcinoma (HNSCC). Analyses were conducted using data from The Cancer Genome Atlas (TCGA) and the Clinical Proteomic Tumor Analysis Consortium (CPTAC). Transcriptomic data from 40 Taiwanese paired OSCC and adjacent nontumor tissue specimens were analyzed using the microarray dataset GSE37991—obtained from the Gene Expression Omnibus (GEO)—to determine whether *HMGA2* expression (probe ID: ILMN _1666236) is associated with OSCC tumorigenesis. *HMGA2* expression and survival data for patients with HNSCC were retrieved from the KM Plotter database (accessed on February 23, 2026), and the prognostic significance of *HMGA2* expression was evaluated through the Kaplan-Meier survival analysis. Finally, potential miRNAs targeting *HMGA2* were identified using data from the miRDB database (http://mirdb.org/).

### Statistical analysis

Demographic variables were compared between cancer-free individuals (healthy controls) and patients with TSCC by using the Mann-Whitney *U* test or Fisher's exact test. Logistic regression models were used to calculate odds ratios (ORs) and 95% confidence intervals (CIs) to identify the associations of genotype frequency with TSCC susceptibility and clinicopathological parameters. Betel quid chewing, cigarette smoking, and alcohol consumption were included as covariates in the adjusted analyses. All statistical analyses were conducted using SAS (version 9.1; SAS Institute, San Francisco, CA, USA).

## Results

### General characteristics of the study cohort

In this study, we examined the association between *HMGA2* polymorphisms and TSCC clinicopathological features by analyzing the data of 383 Taiwanese men with TSCC (TSCC group) and 911 cancer-free individuals (control group). Their demographic and lifestyle characteristics are summarized in Table 1. Age distribution was similar between the TSCC and control groups. Analysis of lifestyle data revealed that the prevalence of betel quid chewing, alcohol consumption, and cigarette smoking was significantly higher in the TSCC group than in the control group. These findings are consistent with those from other Asian OSCC cohorts 23, 24, highlighting the aforementioned lifestyle behaviors as major environmental risk factors for TSCC carcinogenesis. Clinically, most patients with TSCC exhibited no LN involvement (60.8%) or distant metastasis (99.5%), and most tumors exhibited moderate to poor differentiation (88.8%).

### Effects of *HMGA2* variants on TSCC susceptibility

To determine whether the selected *HMGA2* SNPs (rs6581658 A>G, rs10573247 T>del, rs968697 T>C, and rs8756 A>C) were associated with TSCC risk, adjusted ORs (AORs) with 95% CIs were calculated using multiple logistic regression models adjusted for betel quid chewing, cigarette smoking, and alcohol consumption. The distributions of *HMGA2* genotypes revealed that the most frequent alleles were homozygous A/A for the rs6581658 and rs8756 loci and homozygous T/T for the rs10573247 and rs968697 loci (Table 2). Unlike corresponding wild-type genotypes, *HMGA2* SNPs exhibited no significant difference in frequency between the TSCC and control groups.

### Effects of *HMGA2* variants on TSCC clinicopathological features

To assess the clinical relevance of *HMGA2* polymorphisms in TSCC, we examined the associations between *HMGA2* SNPs and clinicopathological features, including primary tumor size, clinical stage, LN metastasis, and histopathological grade (Tables 3 and 4). Patients carrying the rs6581658 minor allele (AG or GG) were at significantly higher risks of presenting with an advanced clinical stage (stage III or IV; AOR = 1.661, 95% CI = 1.086-2.541, *p* = 0.019) and large primary tumors (>T2; AOR = 1.556, 95% CI = 1.025-2.362, *p* = 0.038) than were those carrying the wild-type AA genotype (Table 3). These findings suggest that rs6581658 facilitates tumor aggressiveness by promoting tumor growth or increasing aggressiveness in TSCC. Patients carrying the rs10573247 deletion polymorphism (TT/Del or Del/Del) exhibited a trend toward an increased risk of LN metastasis (*p* = 0.077; Table 3). By contrast, patients carrying the rs968697 minor allele (TC or CC) exhibited a protective trend against advanced clinical stage (*p* = 0.074) and LN metastasis (*p* = 0.079; Table 4).

### Influence of environmental factors on the association between *HMGA2* variants and TSCC clinicopathological features

Cigarette smoking and betel quid chewing are well-established risk factors for OSCC. Exposure to these carcinogens can induce distinct molecular changes and alter gene expression profiles in the oral epithelium, thereby dysregulating the expression of various proteins and biomolecules 25. In the present study, we examined whether betel quid chewing and cigarette smoking modify the association between *HMGA2* SNPs and TSCC clinicopathological features. Stratified analysis revealed that among patients who neither chewed betel quid nor smoked, those carrying ≥1 minor allele of rs968697 were at a significantly reduced risk of LN metastasis or advanced-stage disease. However, this protective effect was not observed in patients exposed to either environmental risk factor (Tables 5 and 6). Conversely, among patients with a history of betel quid chewing or cigarette smoking, those carrying ≥1 rs10573247 deletion allele were at a significantly increased risk of LN metastasis (Tables S1 and S2). These findings underscore the complex interplay between genetic susceptibility and environmental exposure, suggesting that *HMGA2* variants contribute to TSCC aggressiveness in the presence and absence of traditional risk factors. Further research is required to elucidate the biological mechanisms underlying these exposure-stratified associations. Insights from such research may inform personalized prevention strategies and targeted therapeutic approaches for genetically susceptible individuals.

### Correlations of upregulated *HMGA2* expression in HNSCC tissues with tumor aggressiveness and poor prognosis

*HMGA2* polymorphisms influence gene expression in tumor cells 19. In this study, we analyzed the TCGA-HNSCC and CPTAC-HNSCC datasets to respectively evaluate HMGA2 mRNA and protein expression in normal and HNSCC tissues and investigate its potential association with tumor aggressive tumor characteristics and prognosis. Expression levels of HMGA2 mRNA (Fig. 1A) and protein (Fig. 1B) were significantly higher in HNSCC tissues than in noncancerous tissues. Consistent with these findings, analysis of the GSE37991 dataset from the GEO database revealed that *HMGA2* expression was markedly higher in OSCC specimens than in corresponding matched normal tissues from a Taiwanese OSCC N/T paired cohort with betel quid chewing and cigarette smoking habits (Fig. 1C). Furthermore, the levels of both HMGA2 transcript (Fig. 1D) and protein (Fig. 1E) were higher in patients with high-grade tumors (grades 2 or 3) than in those with low-grade tumors (grade 1). Kaplan-Meier survival analysis indicated that higher *HMGA2* expression was associated with shorter overall survival in patients with HNSCC (Fig. 1F). Together, these findings indicate that *HMGA2* SNPs are associated with TSCC aggressiveness, and that elevated HMGA2 expression is also correlated with tumor aggressiveness and poor prognosis in TSCC.

## Discussion

HMGA2 plays key roles in multiple aspects of cancer development, including cell proliferation 26, protection against apoptosis 27, cancer stemness 28, angiogenesis 9, metastasis mediated by EMT 29, and drug resistance 11. Upregulated *HMGA2* expression has been observed in various malignancies, including bladder 30, brain 31, colorectal 32, and breast 33 cancers, and is often associated with poor prognosis. The present study revealed that *HMGA2* expression was significantly upregulated in HNSCC and OSCC cohorts and was positively associated with advanced tumor grades and poor prognosis in patients with HNSCC, supporting the oncogenic role of HMGA2 in HNSCC clinicopathological aggressiveness. OSCC is a complex disease driven by multiple genetic alterations 34. Genetic variations such as SNPs may alter gene function and influence disease susceptibility, thereby contributing to increased cancer risk or distinct pathological characteristics. Several genetic polymorphisms have been associated with OSCC susceptibility and aggressive tumor characteristics, often in conjunction with environmental risk factors 35-37. Although elevated *HMGA2* expression contributes to OSCC aggressiveness 9, the potential interaction effects of environmental risk factors and *HMGA2* polymorphisms on OSCC aggressiveness remain largely unclear. To the best of our knowledge, the present study is the first to demonstrate that *HMGA2* polymorphisms are significantly associated with TSCC clinicopathological characteristics in Taiwanese patients with different lifestyle-related risk factors.

We found that carriers of the mutant G allele of rs6581658 (AG+GG) were at significantly increased risks of large tumors (>T2) and advanced clinical stage (III or IV). Among patients who neither chewed betel quid nor smoked, those carrying the rs968697 mutant C allele (TC+CC) were at significantly reduced risks of advanced clinical stage or LN metastasis. Because rs6581658 and rs968697 are located in the 5ʹ near-gene region, the nucleotide changes introduced by these SNPs may alter transcription factor-binding sites, thereby regulating *HMGA2* transcription. The rs968697 CC genotype is associated with low *HMGA2* expression and low high-stage tumor frequency in patients with colorectal cancer 19. Moreover, the rs968697 TC/CC genotypes are strongly associated with a reduced risk of hepatoblastoma in men 20. Furthermore, the rs968697 C allele was significantly associated with reduced susceptibility to gastric cancer (GC). Patients with GC carrying the C allele were less likely to present with advanced-stage disease (stage III or IV) and have longer overall survival than did those not carrying this allele. Consistently, GC tissues carrying the rs968697 TC and CC genotypes exhibited low levels of *HMGA2* mRNA expression 38. Taken together, the results of our study are consistent with those of the aforementioned studies, suggesting that the C allele of rs968697 downregulates *HMGA2* expression in TSCC and subsequently inhibits disease aggressiveness in patients with TSCC. In addition to rs968697, the rs6581658 AG/GG genotypes has been associated with an increased risk of glioma in Chinese children 18. However, whether rs6581658 polymorphisms affect *HMGA2* mRNA expression remains unclear.

The deletion polymorphism rs10573247, located in the 3′ UTR region of *HMGA2*, has been identified as a regulatory variant that may influence mRNA stability and miRNA binding and modulate *HMGA2* expression. A study demonstrated that miR-3125 exhibits a lower binding affinity for the DelTT allele than for the TT allele, whereas miR-4476 exhibits a higher binding affinity for the DelTT allele than for the TT allele 17. Individuals carrying the TT/Del or Del/Del genotypes are at a significantly higher risk of breast cancer than are those carrying the TT/TT genotype 17. Consistent with these findings, our results indicated that patients with TSCC carrying the rs10573247 deletion polymorphism (TT/Del or Del/Del), particularly those who smoked cigarettes and chewed betel quid, had a significantly increased risk of LN metastasis. Exposure to tobacco smoke or betel quid can lead to the widespread dysregulation of miRNAs in HNSCC 25, 39. For instance, treatment with areca nut extract or arecoline has been demonstrated to upregulate several miRNAs (miR-23a, miR-26a, miR-26b, miR-30a-5p, miR-143, and miR-1915) and downregulate others (miR-886-3p, miR-1914, and miR-1305) in human oral fibroblasts. Betel quid chewing has been associated with miR-23a overexpression in patients with OSCC 40. Notably, several of the aforementioned miRNAs (miR-23a, miR-26a, miR-26b, miR-143, and miR-1305) are predicted to target *HMGA2* according to the miRNA target prediction database miRDB 41 (Table 7). Together, these findings suggest that the rs10573247 deletion polymorphism influences the binding of betel-quid-regulated miRNAs, thereby modulating HMGA2 expression and promoting TSCC aggressiveness. Nonetheless, this hypothesis and the precise mechanisms underlying this interaction require further investigation.

Our study has several limitations that should be acknowledged. First, the analyzed cohorts differed in their ethnic composition. All TSCC cases included in the SNP analysis were derived from a Taiwanese population, whereas the clinicopathological associations of HMGA2 expression were evaluated using the TCGA-HNSCC and CPTAC-HNSCC cohorts, which predominantly consist of White/Caucasian individuals. Although the GSE37991 dataset was included as an independent validation cohort, it comprised only 40 Taiwanese OSCC specimens. Therefore, additional validation in larger independent Taiwanese cohorts is warranted to strengthen the clinical relevance and generalizability of our findings. Second, environmental risk factors—specifically betel quid chewing, smoking, and alcohol consumption—were evaluated as binary variables due to the lack of detailed quantitative data on exposure duration, intensity, and former versus current status. Consequently, potential dose-response interactions between lifestyle factors and *HMGA2* polymorphisms could not be analyzed.

Third, although we identified potential associations between the candidate SNPs and various clinicopathological parameters, including clinical stage, tumor size (T stage), and lymph node metastasis (N status), these parameters are biologically and clinically interconnected. Because overall clinical stage is intrinsically determined by T and N categories, treating them as entirely independent endpoints may overstate the consistency of our findings. Thus, these clinicopathological evaluations might be regarded as secondary endpoints, and the observed secondary associations must be interpreted with caution until validated through comprehensive multivariable models in larger independent cohorts. In addition, although significant associations were identified, multiple testing was not formally adjusted in the present exploratory study. Therefore, some associations may represent false-positive findings arising from multiple comparisons. These results should be regarded as hypothesis-generating and require confirmation in independent cohorts.

Fourth, future studies should perform genotype-expression analyses, such as expression quantitative trait locus (eQTL) analyses, using matched DNA and RNA samples from the same TSCC patients to more accurately determine the effects of *HMGA2* polymorphisms on *HMGA2* expression. Such samples would also facilitate the investigation of alterations in miRNA expression associated with different lifestyle-related risk factors. Moreover, future studies involving allele-specific binding assay and luciferase reporter assay are warranted to determine whether the rs10573247 deletion polymorphism alters the binding affinity of these miRNAs to the *HMGA2* 3'UTR and consequently contributes to dysregulated HMGA2 expression. Finally, the lack of long-term follow-up data precluded an assessment of the prognostic significance of *HMGA2* polymorphisms. Future studies with extended clinical follow-up are therefore needed to clarify their associations with patient survival and clinical outcomes.

## Conclusions

Although HMGA2 has been implicated in cancer development, the impacts of *HMGA2* genetic variation in OSCC remain largely unexplored. The novelty of the present study lies not only in identifying distinct allelic effects of rs6581658 on the clinicopathological characteristics of TSCC in a Taiwanese population, but also, importantly, in revealing exposure-stratified associations between *HMGA2* polymorphisms and lifestyle-related risk factors. Specifically, rs968697 and rs10573247 were associated with TSCC aggressiveness among individuals with different exposure profiles, including betel quid chewing and cigarette smoking. These findings extend the current understanding of *HMGA2* genetic variation in TSCC by suggesting that lifestyle-related exposures may modify the associations between *HMGA2* polymorphisms and TSCC progression.

## Supplementary Material

Supplementary tables.

## Figures and Tables

**Figure 1:**
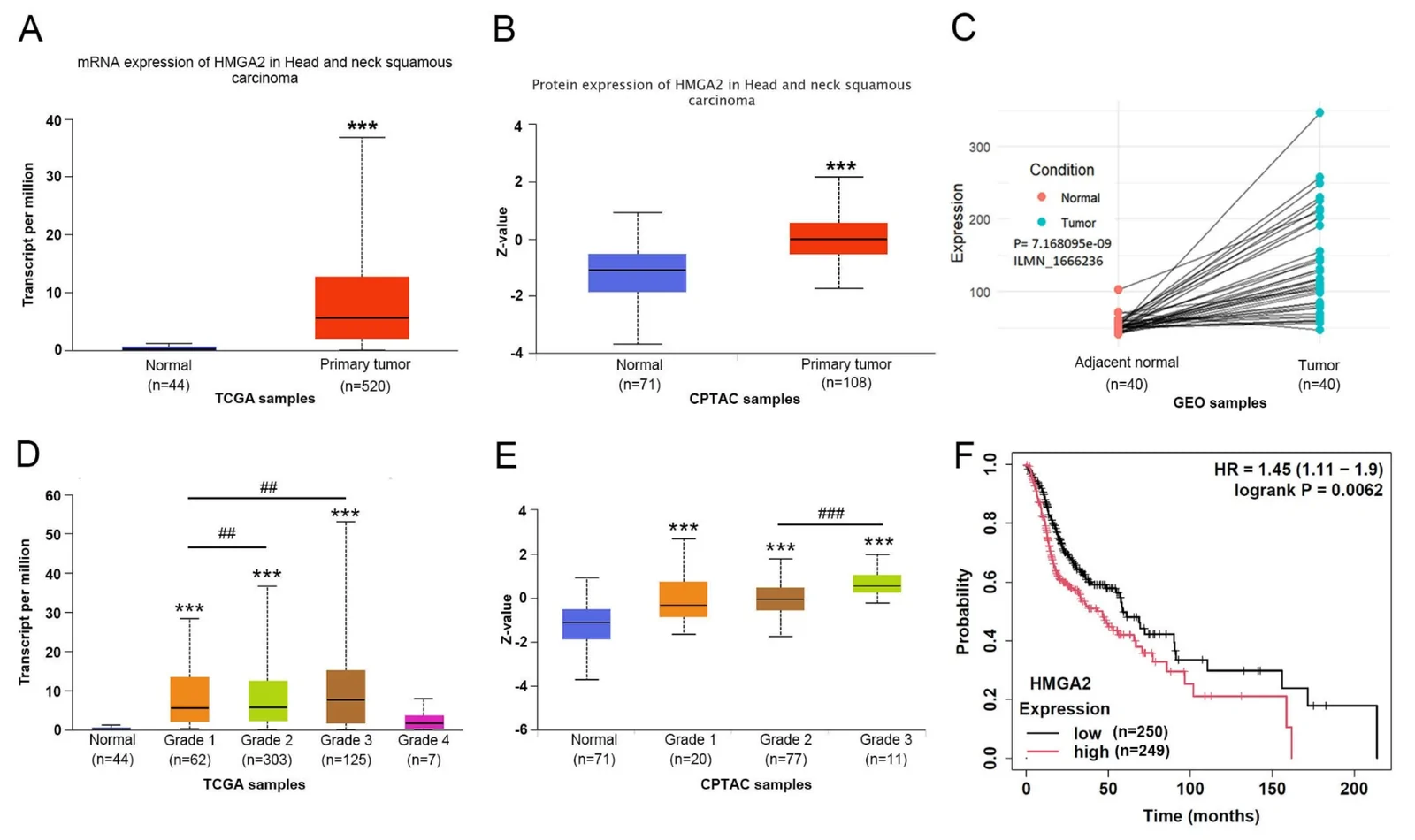
Clinical relevance of *HMGA2* expression in patients with HNSCC. Analyses were performed using data from TCGA, CPTAC, and the GEO databases. Expression levels of HMGA2 (A) gene and (B) protein in normal and HNSCC tissues, analyzed using data from TCGA and the CPTAC. Statistical significance was determined using an unpaired *t* test. (C) *HMGA2* gene expression levels in an OSCC normal/tumorous (N/T) paired cohort with the habits of betel quid chewing and smoking (GSE37991). Statistical significance was determined using a paired *t*-test. *HMGA2* gene (D) and protein (E) expression in tumor tissues from patients with HNSCC stratified by tumor grade. Statistical significance was determined using an unpaired *t* test. Significance: ^***^p < 0.001 versus the control group and ^##^*p* < 0.01 and ^###^*p* < 0.001 versus patients with grade 1 or 2 tumors. (F) The correlation between HMGA2 expression and overall survival in patients with HNSCC was evaluated using the Kaplan-Meier Plotter database. Gene expression levels were dichotomized into high and low groups by using the median value as the cutoff. HR, hazard ratio; HNSCC, head and neck squamous cell carcinoma; TCGA, The Cancer Genome Atlas; GEO, Gene Expression Omnibus; CPTAC, Clinical Proteomic Tumor Analysis Consortium.

**Table 1:** Demographic characteristics of the study cohort.

Variable	Controls (*N* = 911), *n* (%)	Patients (*N* = 383), *n* (%)	*p*
Age (years)			
<60	587 (64.4)	245 (64.0)	0.873
≥60	324 (35.6)	138 (36.0)	
Betel quid chewing			
No	749 (82.2)	130 (34.0)	
Yes	162 (17.8)	253 (66.1)	<0.001^*^
Cigarette smoking			
No	402 (44.1)	88 (23.0)	
Yes	509 (55.9)	295 (77.0)	<0.001^*^
Alcohol consumption			
No	724 (79.5)	238 (62.1)	
Yes	187 (20.5)	145 (37.9)	<0.001^*^
Disease stage			
I+II		169 (44.1)	
III+IV		214 (55.9)	
Tumor T status			
T1+T2		196 (51.2)	
T3+T4		187 (48.8)	
LN status			
N0		233 (60.8)	
N1+N2+N3		150 (39.2)	
Metastasis			
M0		381 (99.5)	
M1		2 (0.5)	
Cell differentiation			
Well differentiated		43 (11.2)	
Moderately or poorly differentiated		340 (88.8)	

^*^p < 0.05 indicates statistical significance.

**Table 2:** Association between *HMGA2* genotypic frequency and TSCC risk

Variable	Controls (*N* = 911), *n* (%)	Patients (*N* = 383), *n* (%)	AOR (95% CI)	*p*
**rs6581658**				
AA	562 (61.7)	236 (61.6)	1.000 (reference)	
AG	306 (33.6)	134 (35.0)	1.213 (0.908-1.621)	0.191
GG	43 (4.7)	13 (3.4)	0.800 (0.390-1.641)	0.543
AG+GG	349 (38.3)	147 (38.4)	1.161 (0.878-1.536)	0.296
**rs10573247**				
TT/TT	574 (63.0)	246 (64.2)	1.000 (reference)	
TT/Del	302 (33.2)	121 (31.6)	0.983 (0.733-1.318)	0.910
Del/Del	35 (3.8)	16 (4.2)	1.134 (0.565-2.276)	0.723
TT/Del+Del/Del	337 (37.0)	137 (35.8)	0.999 (0.753-1.324)	0.993
**rs968697**				
TT	699 (76.7)	289 (75.5)	1.000 (reference)	
TC	203 (22.3)	90 (23.5)	1.085 (0.786-1.498)	0.619
CC	9 (1.0)	4 (1.0)	1.768 (0.486-6.436)	0.387
TC+CC	212 (23.3)	94 (24.5)	1.108 (0.807-1.521)	0.526
**rs8756**				
AA	759 (83.3)	327 (85.4)	1.000 (reference)	
AC	147 (16.1)	52 (13.6)	0.844 (0.574-1.241)	0.389
CC	5 (0.6)	4 (1.0)	2.208 (0.486-10.021)	0.305
AC+CC	152 (16.7)	56 (14.6)	0.885 (0.607-1.288)	0.522

AORs and 95% CIs were estimated using multiple logistic regression models adjusted for betel quid chewing, cigarette smoking, and alcohol consumption. TSCC, tongue squamous cell carcinoma; OR, odds ratio; CI, confidence interval; AOR, adjusted odds ratio.

**Table 3:** Clinical characteristics and genotypic frequencies of *HMGA2* rs6581658 and rs10573247 in patients with TSCC

	rs6581658 (*N* = 383)				rs10573247 (*N* = 383)			
Variable	AA (*N* = 236), *n* (%)	AG+GG (*N* = 147), *n* (%)	AOR (95% CI)	*p*	TT/TT (*N* = 246), *n* (%)	TT/Del + Del/Del (*N* = 137), *n* (%)	AOR (95% CI)	*p*
**Clinical stage**								
I+II	115 (48.7)	54 (36.7)	1.000 (reference)	0.019^*^	114 (46.3)	55 (40.1)	1.000 (reference)	0.244
III+IV	121 (51.3)	93 (63.3)	**1.661 (1.086-2.541)**		132 (53.7)	82 (59.9)	1.287 (0.842-1.966)	
**Tumor size**								
≤T2	131 (55.5)	65 (44.2)	1.000 (reference)	0.038^*^	128 (52.0)	68 (49.6)	1.000 (reference)	0.662
>T2	105 (44.5)	82 (55.8)	1.556 (1.025-2.362)		118 (48.0)	69 (50.4)	1.098 (0.722-1.669)	
**LN metastasis**								
No	149 (63.1)	84 (57.1)	1.000 (reference)	0.244	158 (64.2)	75 (54.7)	1.000 (reference)	0.077
Yes	87 (36.9)	63 (42.9)	1.287 (0.841-1.969)		88 (35.8)	62 (45.3)	1.471 (0.960-2.255)	
**Cell differentiation**								
Well differentiated	22 (9.3)	21 (14.3)	1.000 (reference)	0.128	29 (11.8)	14 (10.2)	1.000 (reference)	0.652
Moderately or poorly differentiated	214 (90.7)	126 (85.7)	0.608 (0.320-1.155)		217 (88.2)	123 (89.8)	1.168 (0.594-2.296)	

AORs and 95% CIs were estimated using multiple logistic regression models adjusted for betel quid chewing, cigarette smoking and alcohol consumption. ^*^p < 0.05 indicates statistical significance. TSCC, tongue squamous cell carcinoma; AOR, adjusted odds ratio; CI, confidence interval.

**Table 4:** Clinical characteristics and genotypic frequencies of *HMGA2* rs968697 and rs8756 in patients with TSCC

	rs968697 (*N* = 383)				rs8756 (*N* = 383)			
Variable	TT (*N* = 289), *n* (%)	TC+CC (*N* = 94), *n* (%)	AOR (95% CI)	*p*	AA (*N* = 327), *n* (%)	AC+CC (*N* = 56), *n* (%)	AOR (95% CI)	*p*
**Clinical stage**								
I+II	120 (41.5)	49 (52.1)	1.000 (reference)	0.074	149 (45.6)	20 (35.7)	1.000 (reference)	0.181
III+IV	169 (58.5)	45 (47.9)	0.651 (0.407-1.042)		178 (54.4)	36 (64.3)	1.496 (0.830-2.699)	
**Tumor size**								
≤T2	145 (50.2)	51 (54.3)	1.000 (reference)	0.448	166 (50.8)	30 (53.6)	1.000 (reference)	0.727
>T2	144 (49.8)	43 (45.7)	0.834 (0.521-1.334)		161 (49.2)	26 (46.4)	0.903 (0.511-1.597)	
**LN metastasis**								
No	169 (58.5)	64 (68.1)	1.000 (reference)	0.079	204 (62.4)	29 (51.8)	1.000 (reference)	0.135
Yes	120 (41.5)	30 (31.9)	0.640 (0.389-1.053)		123 (37.6)	27 (48.2)	1.550 (0.873-2.751)	
**Cell differentiation**								
Well differentiated	33 (11.4)	10 (10.6)	1.000 (reference)	0.859	35 (10.7)	8 (14.3)	1.000 (reference)	0.438
Moderately or poorly differentiated	256 (88.6)	84 (89.4)	1.071 (0.504-2.275)		292 (89.3)	48 (85.7)	0.720 (0.315-1.650)	

AORs and 95% CIs were estimated using multiple logistic regression models adjusted for betel quid chewing, cigarette smoking and alcohol consumption. TSCC, tongue squamous cell carcinoma; AOR, adjusted odds ratio; CI, confidence interval.

**Table 5:** Clinical characteristics and genotypic frequencies of *HMGA2* rs968697 in patients with TSCC stratified by betel-quid-chewing status

	Nonchewers (*N* = 130)				Chewers (*N* = 253)			
Variable	TT (*N* = 95), *n* (%)	TC+CC (*N* = 35), *n* (%)	AOR (95% CI)	*p*	TT (*N* = 194), *n* (%)	TC+CC (*N* = 59), *n* (%)	AOR (95% CI)	*p*
**Clinical stage**								
I+II	36 (37.9)	20 (57.1)	1.000 (reference)	0.062	84 (43.3)	29 (49.2)	1.000 (reference)	0.392
III+IV	59 (62.1)	15 (42.9)	0.462 (0.206-1.039)		110 (56.7)	30 (50.8)	0.774 (0.430-1.392)	
**Tumor size**								
≤T2	47 (49.5)	20 (57.1)	1.000 (reference)	0.410	98 (50.5)	31 (52.5%)	1.000 (reference)	0.721
>T2	48 (50.5)	15 (42.9)	0.714 (0.320-1.591)		96 (49.5)	28 (47.5%)	0.899 (0.499-1.617)	
**LN metastasis**								
No	47 (49.5)	26 (74.3)	1.000 (reference)	0.016^*^	122 (62.9)	38 (64.4)	1.000 (reference)	0.751
Yes	48 (50.5)	9 (25.7)	**0.345 (0.145-0.823)**		72 (37.1)	21 (35.6)	0.905 (0.490-1.674)	
**Cell differentiation**								
Well differentiated	11 (11.6)	3 (8.6)	1.000 (reference)	0.586	22 (11.3)	7 (11.9)	1.000 (reference)	0.865
Moderately or poorly differentiated	84 (88.4)	32 (91.4)	1.458 (0.375-5.674)		172 (88.7)	52 (88.1)	0.924 (0.373-2.293)	

AORs and 95% CIs were estimated using multiple logistic regression models adjusted for cigarette smoking and alcohol consumption. ^*^p < 0.05 indicates statistical significance. TSCC, tongue squamous cell carcinoma; AOR, adjusted odds ratio; CI, confidence interval.

**Table 6:** Clinical characteristics and genotypic frequencies of *HMGA2* rs968697 in patients with TSCC stratified by cigarette smoking status

	Nonsmokers (*N* = 88)				Smokers (*N* = 295)			
Variable	TT (*N* = 59), *n* (%)	TC+CC (*N* = 29), *n* (%)	AOR (95% CI)	*p*	TT (*N* = 230), *n* (%)	TC+CC (*N* = 65), *n* (%)	AOR (95% CI)	*p*
**Clinical stage**								
I+II	21 (35.6)	18 (62.1)	1.000 (reference)	0.020^*^	99 (43.0)	31 (47.7)	1.000 (reference)	0.409
III+IV	38 (64.4)	11 (37.9)	**0.333 (0.132-0.843)**		131 (57.0)	34 (52.3)	0.835 (0.480-1.452)	
**Tumor size**								
≤T2	28 (47.5)	15 (51.7)	1.000 (reference)	0.717	117 (50.9)	36 (55.4)	1.000 (reference)	0.510
>T2	31 (52.5)	14 (48.3)	0.848 (0.347-2.072)		113 (49.1)	29 (44.6)	0.830 (0.477-1.445)	
**LN metastasis**								
No	27 (45.8)	22 (75.9)	1.000 (reference)	0.008^*^	142 (61.7)	42 (64.6)	1.000 (reference)	0.720
Yes	32 (54.2)	7 (24.1)	**0.248 (0.089-0.693)**		88 (38.3)	23 (35.4)	0.900 (0.505-1.603)	
**Cell differentiation**								
Well differentiated	7 (11.9)	2 (6.9)	1.000 (reference)	0.500	26 (11.3)	8 (12.3)	1.000 (reference)	0.826
Moderately or poorly differentiated	52 (88.1)	27 (93.1)	1.767 (0.338-9.240)		204 (88.7)	57 (87.7)	0.909 (0.390-2.120)	

AORs and 95% CIs were estimated using multiple logistic regression models adjusted for betel quid chewing, and alcohol consumption. ^*^p < 0.05 indicates statistical significance. TSCC, tongue squamous cell carcinoma; AOR, adjusted odds ratio; CI, confidence interval.

**Table 7:** ANE- or ARE-regulated *HMGA2*-targeting miRNAs predicted using the miRDB database

Target score	miRNA name	Target gene symbol	Differential expression after ANE or ARE treatment
96	hsa-miR-1305	*HMGA2*	Downregulation
92	hsa-miR-26a-5p	*HMGA2*	Upregulation
92	hsa-miR-26b-5p	*HMGA2*	Upregulation
92	hsa-miR-143-5p	*HMGA2*	Upregulation
67	hsa-miR-23a-3p	*HMGA2*	Upregulation

ANE, areca nut extract; ARE, arecoline

## Data Availability

The data supporting the findings of this study are available within the article and its supplementary materials. Additional original data are available from the corresponding author upon reasonable request.

## Abbreviations

CIs: Confidence intervals
CPTAC: Clinical Proteomic Tumor Analysis Consortium
EMT: Epithelial-mesenchymal transition
GEO: Gene Expression Omnibus
HMGA2: High-mobility group A protein 2
HNSCC: Head and neck squamous cell carcinoma
miRNA: microRNA
OR: Odds ratio
OSCC: Oral squamous cell carcinoma
SNPs: Single-nucleotide polymorphisms
TCGA: The Cancer Genome Atlas
TSCC: Tongue squamous cell carcinoma
UTR: Untranslated region
