# Supplementary material for: Associations of HMGA2 single-nucleotide polymorphisms with clinicopathological features of tongue squamous cell carcinoma in Taiwanese men with distinct lifestyle-related risk factors
Source: J Cancer. 2026 Sep 11;17(10):1709–19. doi: 10.7150/jca.139096 (PMC13617415; doi:10.7150/jca.139096)
Supplement: Supplementary file 1 [file jcav17p1709s1.pdf]

Supplemental Table 1. Clinical characteristics and genotypic frequencies of *HMGA2* rs10573247 in patients with TSCC stratified by betel-quid-chewing status

	Nonchewers (*N* = 130)				Chewers (*N* = 253)			
Variable	TT/TT (*N*=82), *n* (%)	TT/Del + Del/Del (*N*=48), *n* (%)	OR (95% CI)	*p*	TT/TT (*N*=164), *n* (%)	TT/Del + Del/Del (*N*=89), *n* (%)	OR (95% CI)	*p*
**Clinical stage**								
I+II	37 (45.1)	19 (39.6)	1.000 (reference)	0.538	77 (47.0)	36 (40.4)	1.000 (reference)	0.321
III+IV	45 (54.9)	29 (60.4)	1.255 (0.609~2.588)		87 (53.0)	53 (59.6)	1.303 (0.773~2.198)	
**Tumor size**								
≤T2	43 (52.4)	24 (50.0)	1.000 (reference)	0.788	85 (51.8)	44 (49.4)	1.000 (reference)	0.716
>T2	39 (47.6)	24 (50.0)	1.103 (0.541~2.249)		79 (48.2)	45 (50.6)	1.100 (0.657~1.844)	
**LN metastasis**								
No	46 (56.1)	27 (56.3)	1.000 (reference)	0.987	112 (68.3)	48 (53.9)	1.000 (reference)	**0.024***
Yes	36 (43.9)	21 (43.7)	0.994 (0.485~2.038)		52 (31.7)	41 (46.1)	**1.840 (1.082~3.129)**	
**Cell differentiation**								
Well differentiated	7 (8.5)	7 (14.6)	1.000 (reference)	0.283	22 (13.4)	7 (7.9)	1.000 (reference)	0.186
Moderately or poorly differentiated	75 (91.5)	41 (85.4)	0.547 (0.179~1.667)		142 (86.6)	82 (92.1)	1.815 (0.743~4.432)	

ORs and 95% CIs were estimated using logistic regression models.

*$p < 0.05$ indicates statistical significance.

TSCC, tongue squamous cell carcinoma; OR, odds ratio; CI, confidence interval.

Supplemental Table 2. Clinical characteristics and genotypic frequencies of HMGA2 rs10573247 in patients with TSCC stratified by cigarette smoking status

	Nonsmoker (*N*=88)				Smoker (*N*=295)			
Variable	TT/TT (N=54), *n* (%)	TT/Del + Del/Del (N=34), *n* (%)	OR (95% CI)	*p*	TT/TT (N=192), *n* (%)	TT/Del + Del/Del (*N*=103), *n* (%)	OR (95% CI)	*p*
**Clinical stage**								
I+II	24 (44.4)	15 (44.1)	1.000 (reference)	0.976	90 (46.9)	40 (38.8)	1.000 (reference)	0.185
III+IV	30 (55.6)	19 (55.9)	1.013 (0.427~2.404)		102 (53.1)	63 (61.2)	1.390 (0.854~2.262)	
**Tumor size**								
≤T2	27 (50.0)	16 (47.1)	1.000 (reference)	0.788	101 (52.6)	52 (50.5)	1.000 (reference)	0.728
>T2	27 (50.0)	18 (52.9)	1.125 (0.476~2.656)		91 (47.4)	51 (49.5)	1.089 (0.674~1.757)	
**LN metastasis**								
No	27 (50.0)	22 (64.7)	1.000 (reference)	0.176	131 (68.2)	53 (51.5)	1.000 (reference)	**0.005***
Yes	27 (50.0)	12 (35.3)	0.545 (0.226~1.319)		61 (31.8)	50 (48.5)	**2.026 (1.239~3.312)**	
**Cell differentiation**								
Well differentiated	6 (11.1)	3 (8.8)	1.000 (reference)	0.730	23 (12.0)	11 (10.7)	1.000 (reference)	0.739
Moderately or poorly differentiated	48 (88.9)	31 (91.2)	1.292 (0.301~5.549)		169 (88.0)	92 (89.3)	1.138 (0.531~2.439)	

ORs and 95% CIs were estimated using logistic regression models.

[*] $p < 0.05$ indicates statistical significance.

TSCC, tongue squamous cell carcinoma; OR, odds ratio; CI, confidence interval.
